# Supplementary material for: Estimation of Ecological and Human Health Risks Posed by Heavy Metals in Street Dust of Madrid City (Spain)
Source: Int J Environ Res Public Health. 2022 Apr 26;19(9):5263. doi: 10.3390/ijerph19095263 (PMC9105467; doi:10.3390/ijerph19095263)
Supplement: Supplementary file 1 [file ijerph-19-05263-s001.zip › ijerph-1651832-supplementary.pdf]

Table S1: Coordinates of urban dust sampling sites

Sample	Latitude	Longitude
(0,1)	40°24´29,39´´	3°42´45,68´´
(0,2)	40°24´44,34´´	3° 42´45,13´´
(0,3)	40°24´59,02´´	3° 42´46,91´´
(0,4)	40° 25´14,88´´	3° 42´45,30´´
(0,5)	40°25´26,08´´	3° 42´42,80´´
(0,6)	40°25´41,68´´	3°42´47,85´´
(0,7)	40°25´59,71´´	3°42´45,41´´
(1,1)	40°24´28,65´´	3°42´26,49´´
(1,2)	40°24´44,74´´	3°42´26,51´´
(1,3)	40°24´58,57´´	3°42´25,32´´
(1,4)	40°25´13,26´´	3°42´22,89´´
(1,5)	40°25´29,72´´	3°42´26,00´´
(1,6)	40°25´44,38´´	3°42´25,19´´
(1,7)	40°25´58,52´´	3°42´26,24´´
(2,1)	40°24´27,46´´	3°42´10,50´´
(2,2)	40°24´44,00´´	3°42´06,65´´
(2,3)	40°25´00,29´´	3°42´08,25´´
(2,4)	40°25´09,58´´	3°42´00,28´´
(2,5)	40°25´25,35´´	3°42´06,63´´
(2,6)	40°25´43,09´´	3°42´09,18´´
(2,7)	40°25´57,07´´	3°42´06,80´´
(3,1)	40°24´38,82´´	3°42´47,22´´
(3,2)	40°24´45,67´´	3°41´45,31´´
(3,3)	40°25´00,33´´	3°41´44,15´´
(3,4)	40°25´12,11´´	3°41´46,08´´
(3,5)	40°25´27,63´´	3°41´42,86´´
(3,6)	40°26´42,30´´	3°41´44,19´´
(3,7)	40°26´59,50´´	3°41´51,99´´
(4,1)	40°24´27,11´´	3°41´21,40´´
(4,2)	40°24´45,55´´	3°41´24,82´´
(4,3)	40°24´59,52´´	3°41´24,40´´
(4,4)	40°25´12,41´´	3°41´26,66´´
(4,5)	40°25´30,00´´	3°41´16,84´´
(4,6)	40°25´41,15´´	3°41´27,95´´
(4,7)	40°25´57,69´´	3°41´25,26´´

Table S2. Pearson's correlation matrix between the variables studied.

	Cd CF	Cr CF	Cu CF	Ni CF	Pb CF	Zn CF	PL CF	PL PLI	PL RI	Ca	Cd	Cr	Cu	Ni	Pb	Zn	PL	Cd EF	Cr EF	Cu EF	Ni EF	Pb EF	Zn EF	PL EF	Cd Igeo	Cr Igeo	Cu Igeo	Ni Igeo	Pb Igeo	Zn Igeo	PL Igeo
**Cd CF**	1	-.001	.349*	-.049	.020	.200	.198	.457**	.457**	-.032	1.000**	-.001	.349*	-.049	.020	.200	.198	.856**	.022	.404*	-.033	.058	.248	.303	.936**	.061	.361*	-.027	.299	.217	.281
**Cr CF**	-.001	1	.248	.907**	-.032	.310	.263	.682**	.001	.126	-.002	1.000**	.248	.907**	-.032	.310	.263	-.075	.819**	.132	.753**	-.036	.171	.186	.116	.932**	.353*	.878**	.189	.396*	.455**
**Cu CF**	.349*	.248	1	.146	-.060	.327	.392*	.653**	.138	-.025	.348*	.248	1.000**	.145	-.060	.327	.392*	.344*	.213	.930**	.128	-.040	.272	.445**	.469**	.345*	.914**	.318	.092	.400*	.511**
**Ni CF**	-.049	.907**	.146	1	-.069	.192	.154	.540**	-.058	.109	-.049	.907**	.146	1.000**	-.069	.192	.154	-.086	.748**	.079	.856**	-.069	.112	.119	.034	.776**	.214	.887**	.072	.254	.300
**Pb CF**	.020	-.032	-.060	-.069	1	.258	.781**	.254	.897**	.255	.020	-.031	-.060	-.069	1.000**	.258	.781**	-.043	-.105	-.086	-.113	.953**	.070	.433*	.031	-.012	-.080	-.108	.752**	.289	.544**
**Zn CF**	.200	.310	.327	.192	.258	1	.747**	.660**	.343*	-.107	.201	.310	.327	.192	.258	1.000**	.747**	.267	.380*	.364*	.255	.312	.905**	.835**	.261	.422*	.350*	.314	.391*	.947**	.822**
**PL CF**	.198	.263	.392*	.154	.781**	.747**	1	.701**	.806**	.127	.198	.263	.392*	.154	.781**	.747**	1.000**	.180	.223	.366*	.137	.780**	.551**	.813**	.274	.347*	.373*	.225	.730**	.769**	.918**
**PL PLI**	.457**	.682**	.653**	.540**	.254	.660**	.701**	1	.462**	-.003	.457**	.682**	.653**	.540**	.254	.660**	.701**	.410*	.636**	.609**	.513**	.321	.545**	.707**	.593**	.744**	.683**	.652**	.567**	.732**	.848**
**PL RI**	.457**	.001	.138	-.058	.897**	.343*	.806**	.462**	1	.212	.458**	.001	.138	-.058	.897**	.343*	.806**	.340*	-.058	.135	-.093	.873**	.192	.543**	.447**	.048	.124	-.077	.806**	.382*	.638**
**Ca**	-.032	.126	-.025	.109	.255	-.107	.127	-.003	.212	1	-.032	.126	-.025	.109	.255	-.107	.127	-.403*	-.390*	-.323	-.312	.044	-.437**	-.376*	-.144	.064	.050	.037	.040	-.060	.065
**Cd**	1.000**	-.002	.348*	-.049	.020	.201	.198	.457**	.458**	-.032	1	-.002	.348*	-.049	.020	.201	.198	.856**	.021	.404*	-.033	.058	.249	.303	.936**	.060	.361*	-.028	.299	.218	.281
**Cr**	-.001	1.000**	.248	.907**	-.031	.310	.263	.682**	.001	.126	-.002	1	.248	.907**	-.031	.310	.263	-.075	.819**	.132	.753**	-.036	.170	.186	.116	.932**	.353*	.878**	.189	.396*	.455**
**Cu**	.349*	.248	1.000**	.146	-.060	.327	.392*	.653**	.138	-.025	.348*	.248	1	.145	-.060	.327	.392*	.344*	.212	.930**	.128	-.040	.272	.445**	.469**	.345*	.914**	.318	.092	.400*	.511**
**Ni**	-.049	.907**	.145	1.000**	-.069	.192	.154	.540**	-.058	.109	-.049	.907**	.145	1	-.069	.192	.154	-.086	.748**	.079	.856**	-.069	.112	.119	.034	.775**	.214	.887**	.072	.254	.300
**Pb**	.020	-.032	-.060	-.069	1.000**	.258	.781**	.254	.897**	.255	.020	-.031	-.060	-.069	1	.258	.781**	-.043	-.105	-.086	-.113	.953**	.070	.433*	.031	-.012	-.080	-.108	.752**	.289	.544**

	Cd CF	Cr CF	Cu CF	Ni CF	Pb CF	Zn CF	PL CF	PL PLI	PL RI	Ca	Cd	Cr	Cu	Ni	Pb	Zn	PL	Cd EF	Cr EF	Cu EF	Ni EF	Pb EF	Zn EF	PL EF	Cd Igeo	Cr Igeo	Cu Igeo	Ni Igeo	Pb Igeo	Zn Igeo	PL Igeo
Zn	.200	.310	.327	.192	.258	1.000**	.747**	.660**	.343*	-.107	.201	.310	.327	.192	.258	1	.747**	.267	.380*	.365*	.255	.312	.905**	.835**	.261	.422*	.350*	.314	.391*	.947**	.822**
PL	.198	.263	.392*	.154	.781**	.747**	1.000**	.701**	.806**	0.127	0.198	0.263	.392*	.154	.781**	.747**	1	.180	.223	.366*	.137	.780**	.551**	.813**	.274	.347*	.373*	.225	.730**	.769**	.918**
Cd EF	.856**	-.075	.344*	-.086	-.043	.267	.180	.410*	.340*	-.403*	.856**	-.075	.344*	-.086	-.043	.267	.180	1	0.160	.549**	.094	.071	.456**	.491**	.827**	-.009	.318	-.042	.216	.236	.234
Cr EF	.022	.819**	.213	.748**	-.105	.380*	.223	.636**	-.058	-.390*	.021	.819**	.212	.748**	-.105	.380*	.223	.160	1	.277	.900**	.008	.442**	.431*	.161	.822**	.291	.814**	.157	.397*	.394*
Cu EF	.404*	.132	.930**	.079	-.086	.364*	.366*	.609**	.135	-.323	.404*	.132	.930**	.079	-.086	.365*	.366*	.549**	.277	1	.205	-.001	.433*	.588**	.518**	.235	.822**	.242	.091	.404*	.464**
Ni EF	-.033	.753**	.128	.856**	-.113	.255	.137	.513**	-.093	-.312	-.033	.753**	.128	.856**	-.113	.255	.137	.094	.900**	.205	1	-.019	.331	.326	.064	.690**	.176	.847**	.055	.264	.263
Pb EF	.058	-.036	-.040	-.069	.953**	.312	.780**	.321	.873**	.044	.058	-.036	-.040	-.069	.953**	.312	.780**	.071	.008	-.001	-.019	1	.203	.570**	.092	-.004	-.075	-.083	.831**	.335	.574**
Zn EF	.248	.171	.272	.112	.070	.905**	.551**	.545**	.192	-.437**	.249	.170	.272	.112	.070	.905**	.551**	.456**	.442**	.433*	.331	.203	1	.871**	.300	.282	.279	.249	.272	.798**	.632**
PL EF	.303	.186	.445**	.119	.433*	.835**	.813**	.707**	.543**	-.376*	.303	.186	.445**	.119	.433*	.835**	.813**	.491**	.431*	.588**	.326	.570**	.871**	1	.393*	.300	.406*	.250	.574**	.791**	.813**
Cd Igeo	.936**	.116	.469**	.034	.031	.261	.274	.593**	.447**	-.144	.936**	.116	.469**	.034	.031	.261	.274	.827**	.161	.518**	.064	.092	.300	.393*	1	.200	.486**	.097	.377*	.307	.397*
Cr Igeo	.061	.932**	.345*	.776**	-.012	.422*	.347*	.744**	.048	.064	.060	.932**	.345*	.775**	-.012	.422*	.347*	-.009	.822**	.235	.690**	-.004	.282	.300	.200	1	.475**	.890**	.222	.514**	.566**
Cu Igeo	.361*	.353*	.914**	.214	-.080	.350*	.373*	.683**	.124	.050	.361*	.353*	.914**	.214	-.080	.350*	.373*	.318	.291	.822**	.176	-.075	.279	.406*	.486**	.475**	1	.434*	.061	.450**	.561**
Ni Igeo	-.027	.878**	.318	.887**	-.108	.314	.225	.652**	-.077	.037	-.028	.878**	.318	.887**	-.108	.314	.225	-.042	.814**	.242	.847**	-.083	.249	.250	.097	.890**	.434*	1	.053	.372*	.416*
Pb Igeo	.299	.189	.092	.072	.752**	.391*	.730**	.567**	.806**	.040	.299	.189	.092	.072	.752**	.391*	.730**	.216	.157	.091	.055	.831**	.272	.574**	.377*	.222	.061	.053	1	.453**	.681**
Zn Igeo	.217	.396*	.400*	.254	.289	.947**	.769**	.732**	.382*	-.060	.218	.396*	.400*	.254	.289	.947**	.769**	.236	.397*	.404*	.264	.335	.798**	.791**	.307	.514**	.450**	.372*	.453**	1	.903**
PL Igeo	.281	.455**	.511**	.300	.544**	.822**	.918**	.848**	.638**	.065	.281	.455**	.511**	.300	.544**	.822**	.918**	.234	.394*	.464**	.263	.574**	.632**	.813**	.397*	.566**	.561**	.416*	.681**	.903**	1

*. The correlation is significant at the .05 level (bilateral). **. The correlation is significant at the .01 level (bilateral).
